# Surfactant Modified Graphite Paste Electrode as an Electrochemical Sensor for the Enhanced Voltammetric Detection of Estriol with Dopamine and Uric acid

**DOI:** 10.34172/apb.2020.029

**Published:** 2020-02-18

**Authors:** Madikeri Manjunath Charithra, Jamballi Gangadharappa Gowda Manjunatha, Chenthattil Raril

**Affiliations:** Department of Chemistry, FMKMC College, Mangalore University Constituent College, Madikeri, Karnataka, India.

**Keywords:** Electrochemical sensor, Estriol, Graphite paste electrode, Octoxynol-9 immobilization

## Abstract

***Purpose:*** Estriol (ERL) is a type of hormone among the groups of estrogen hormone that was detected through the voltammetric technique by constructing an electrochemical sensor based on the octoxynol-9 modified graphite paste electrode (OXL-9MGPE).

***Methods:*** Using the strategy of cyclic voltammetry (CV) and differential pulse voltammetry (DPV) with a bare graphite paste electrode (BGPE) immobilized with OXL-9, ERL electro-oxidation has been assessed in 0.2 M phosphate buffer solution (PBS) of pH 6.0. The fabricated electrode has substantial electrochemical sensing efficiency, and the ERL oxidation at the OXL-9MGPE was the irreversible process. The surface morphological characteristics of BGPE and OXL-9MGPE were differentiated with the help of field emission scanning electron microscopy (FE-SEM).

***Results:*** The impact of various factors such as scan rate, pH, reproducibility, repeatability, and stability on the electro-oxidation of ERL was evaluated. Techniques of CV and DPV were utilized to determine ERL, dopamine (DAN), and uric acid (URA) simultaneously with the projected sensor. The peak current was varied with ERL concentration in the range from 4×10^-5^ to 1.2×10^-4^ M at OXL-9MGPE. From this, the detection limit 1.4×10^ -6^ M and limit of quantification (LOQ) 4.7×10^-6^ M have been attained.

***Conclusion:*** As a result, OXL-9MGPE was successfully achieved as an electrochemical detector for the electro analysis of ERL via the CV technique.

## Introduction

Estrogens are the hormones present in the ovarian follicles of premenopausal women, ERL has become one of the three principal estrogens (estriol [ERL], estradiol, estrone) produced during pregnancy, and it is secreted mainly by the placenta.^1-3^ It is regarded as one of the essential steroid estrogen, that adversely affects the reproductive and sexual functioning and bone structure. ERL oral tablets were used for the diagnosis of local urogenital problems in postmenopausal women, and it is used to treat the estrogen deficiency symptoms such as sexual disorder.^4-6^ ERL is found to be an organic contaminant in the environmental water, and it is also considered as endocrine disrupting chemical, which affects by interfering with the function of hormones in the body and results to various diseases.^7-8^

The reviewed literature reveals that the many conventional techniques have been established to determine the ERL, mainly chromatographic technique,^9-11^ electrophoresis,^12,13^ immunoassay,^14-16^ UFLC-fluorescence.^17^ Electrochemical approaches are commonly used for the determination of bioactive substances due to their simplicity, excellent sensitivity, inexpensive, and secure handling. The kinetics of heterogeneous electron transmission reactions, redox process thermodynamics, ion transfer processes, and adsorption phenomenon can be studied with the electrochemical methods. The cyclic voltammetry (CV) is also electrochemical techniques in which a redox system can be considered.^18-21^

Octoxynol-9 (OXL-9) is a surface-active agent, which is amphiphilic in nature. Surfactants are widely used in the research field of electrochemistry and electroanalytical chemistry.^22,23^ Surfactants can be absorbed in the interfaces and on the electrode surface and adsorption initiates beneath the critical micelle concentration (CMC). The micelle aggregates considerably vary the charge transfer coefficient, and redox potential due to the adsorption of surfactant on the electrode and solubilisation of the electroactive compound. By immobilization of surfactant, voltammetric responses of analysed moieties were significantly upgraded.^24-26^

**Scheme 1:**
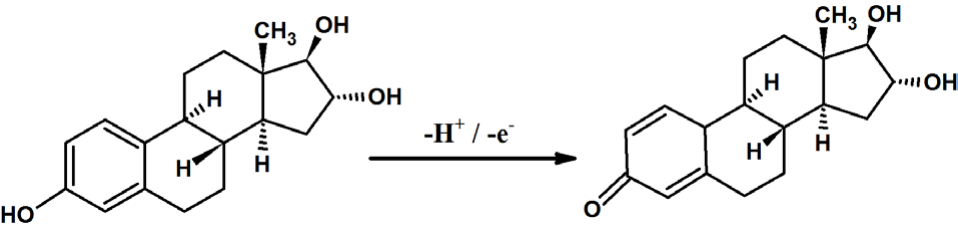
Mechanism of ERL electro-oxidation.

This present work aims at the fabrication of octoxynol-9 modified graphite paste electrode (OXL-9MGPE) for the sensitive detection of ERL, including detection of dopamine (DAN), uric acid (URA), and ERL simultaneously by using CV and differential pulse voltammetry (DPV) technique. OXL-9MGPE enhanced the electrochemical behaviour of ERL. Well-separated peaks were attained for the interference studies of ERL, DAN, and URA indicates the selectivity of the constructed sensor. To the best, the examination of the previous literature revealed that there was no article regarding the electro-oxidation of ERL at OXL-9MGPE by CV technique. Scheme 1 exposes the mechanism of ERL electro-oxidation. Finally, for the electroanalysis of ERL, a simple, low cost, fast response, and sensitive electroanalytical method were developed.

## Materials and Methods

### Instrumentation

Voltammetric measurements (CV and DPV) were performed by using an electrochemical analyser model-201 (EA-201 chemilink system), which is connected to the personal computer. The electrochemical analyser fitted with a typical tri-electrode arrangement. The tri-electrode system involves a bare graphite paste electrode (BGPE) and OXL-9MGPE as the working electrode, Calomel electrode is functioning as the reference electrode, and platinum wire employed as the counter electrode. The potential was given against SCE. The digital pH meter of the EQ-610 model has been used to provide the solution with the appropriate pH for the experiment.

### Reagents and preparations

The ERL was procured from TCI Company Ltd. (Japan). Silicone oil and Graphite powder were acquired from Nice Chemicals, India. NaH_2_PO_4_, Na_2_HPO_4_, OXL-9 were purchased from Himedia chemicals, Bangalore, India. All the chemicals utilized in this electro-analysis of ERL were of analytical quality and used as received. Alcohol was used to prepare the standard ERL solution (25×10^-4^ M). OXL-9 solution (25×10^-4^ M) was prepared with distilled water. Phosphate buffer solution (PBS) (0.2 M) was prepared by adding an appropriate quantity of 0.2 M NaH_2_PO_4_ and 0.2 M Na_2_HPO_4_ and functioning as a supporting electrolyte. All experiments were conducted at the temperature of the laboratory (25±1°C).

### Construction of BGPE

The BGPE was prepared by using the configuration of 70:30 (graphite powder: silicone oil) in an agate mortar and ground with the aid of pestle for about 30 minutes up to a homogenous paste was formed. The Teflon tube owing a cavity was filled with the formed homogenous carbon paste that was smoothened on a tissue paper. Thus, BGPE has been obtained. Copper wire for the purpose of electrical contact connected at the end of the Teflon tube.

### Fabrication of OXL-9MGPE

The OXL-9MGPE was fabricated by adopting the immobilization technique. The OXL-9MGPE was constructed by immobilizing 10 µL solution of OXL-9 on the surface of the above-prepared BGPE and allowed it for about 5 minutes at room temperature. After 5 minutes, the electrode was cleaned with distilled water to eliminate the unabsorbed OXL-9. Thus, OXL-9MGPE was fabricated. The constructed OXL-9MGPE is functioning as a working electrode for the detection of ERL.

## Results and Discussion

### Surface characterization of BGPE and OXL-9MGPE

**Figure 1:**
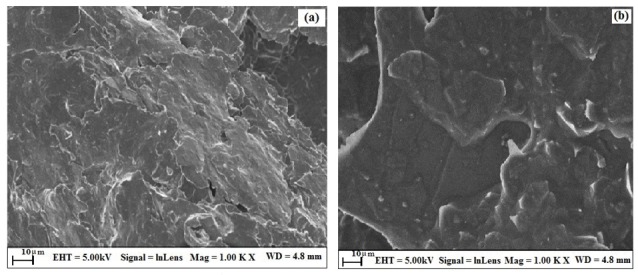
FE-SEM Surface analysis of BGPE (a), OXL-9MGPE (b).

The surface morphology of the thin film was studied by employing field emission scanning electron microscopy (FE-SEM) characterizing technique. The topological features of the surface of BGPE and OXL-9MGPE were explored by FE-SEM. Figure 1a shows the FE-SEM magnified image of BGPE, which indicates that the irregular shape, flakes of graphite. Figure 1b shows the FE-SEM resolution of OXL-9MGPE, which provides that the even film, had smooth surface morphology, which increases the electrode surface area. The adsorption of OXL-9 on the BGPE surface was clearly specified in the FE-SEM image.

### Evaluation of electrochemical behaviour of ERL

**Figure 2:**
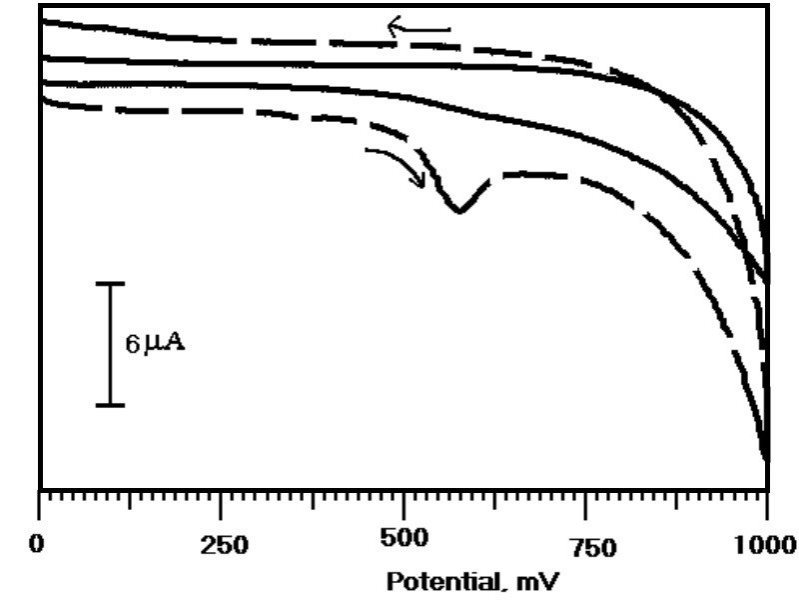
Cyclic voltammograms (CVs) at OXL-9MGPE in the appearance of 1×10^-4^ M ERL (thick broken line) and non-appearance of ERL (thick continuous line) in 0.2 M PBS of pH 6.0, at the potential sweep rate 100 mVs^-1^.

The electrochemical response of the ERL at the OXL-9MGPE was examined in presence and absence of ERL in 0.2 M PBS of pH 6.0 by CV technique. The oxidation of ERL at the OXL-9MGPE with PBS at pH 6.0 with (thick broken line) and without ( thick continuous line) ERL was recorded from 0 to 1000 mV potential window, at the sweep rate of 100 mV/s by using CV was presented in Figure 2. In the existence of 1×10^-4^ M ERL, the oxidation peak potential and the peak current was observed at 576 mV, 6.8 µA respectively, and that oxidation peak was not observed in the nonappearance of ERL. The appearance of the oxidation peak in the existence of ERL approves the electrochemical activity of ERL at OXL-9MGPE.

### Investigation of voltammetric response of ERL at BGPE and OXL-9MGPE by CV method

**Figure 3:**
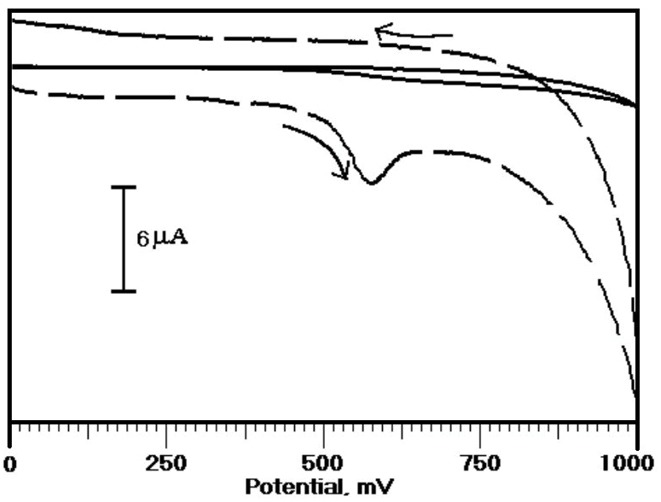
CVs obtained for the voltammetric response of ERL (1×10^-4^ M) at BGPE (thick steady line), and OXL-9MGPE (thick broken line) in 0.2 M PBS of pH 6.0, at the potential scan rate 100 mVs^-1^.

The voltammetric response of the ERL has been assessed with the aid of the CV technique at OXL-9MGPE in 0.2 M PBS of pH 6.0. Figure 3 describes the CVs of the electro-oxidation of ERL at the BGPE (thick steady line) and OXL-9MGPE (thick broken line) in 0.2 M PBS of pH 6.0 at the scan rate 100 mVs^-1^. At BGPE, the oxidation peak was not observed, but at OXL-9MGPE, the peak was obtained for ERL with E_pa_ at 576 mV. The electrochemical response of ERL was good at OXL-9MGPE compared to BGPE. Thus, the above results suggest that the sensing performance of the OXL-9MGPE has successfully enhanced the electro-oxidation of ERL.

### Evaluation of electrochemical characterization of OXL-9MGPE

**Figure 4:**
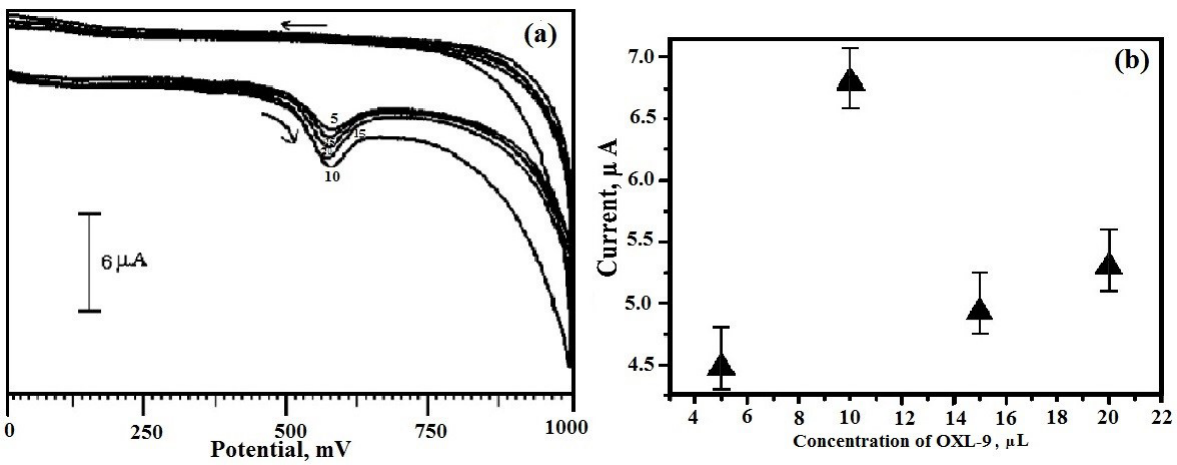
(a) CVs for OXL-9MGPE of ERL (1×10^-4^ M) with varying a concentration range from 5 µL to 20 µL in 0.2 M PBS of pH 6.0, at the scan rate 100 mVs^-1^, (b) The plot of concentration of OXL-9 vs. I_pa._

To fabricate OXL-9MGPE, it was very significant to optimize the OXL-9 solution on the BGPE to boost the electro-oxidation of ERL. The various concentration of OXL-9 solution from the range 5 µL to 20 µL was coated drop wise on the surface of BGPE. The I_pa_ of the oxidation of ERL in 0.2 M PBS of pH 6.0 at OXL-9 MGPE was boosted with the increase in OXL-9 concentration was shown in Figure 4a. After reaching the concentration of 10 µL of OXL-9, the peak current response was supreme, and the obtained peak current for further addition in the concentration of OXL-9 was less than peak current acquired for 10 µL of OXL-9 (Figure 4b). Therefore, 10 µL was preferred for the adsorption of the OXL-9 molecule onto the BGPE.

### Investigation of impact of sweep rate on the electro-oxidation of ERL

**Figure 5:**
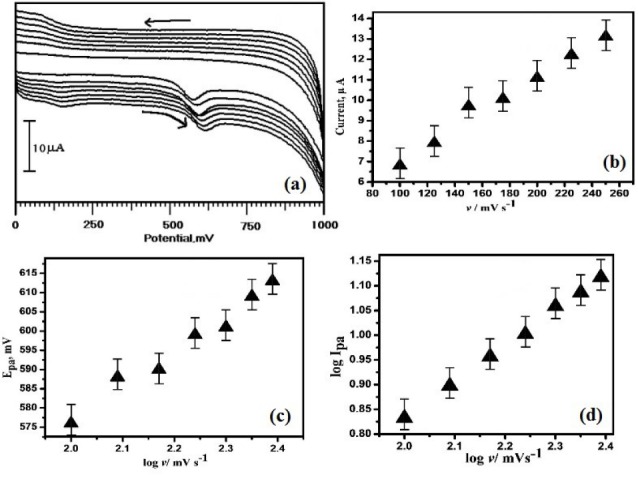
(a) CVs acquired for the voltammetric behaviour of 1× 10^-4^ M ERL at a different sweep rate by using OXL-9MGPE in 0.2 M PBS of pH 6.0, (b) The plot of sweep rate vs. I_pa_, (c) The graphical plot of the log of sweep rate vs. E_pa_, (d) The graph of log sweep rate vs. log I_pa_.

To recognize the electro-oxidation mechanism of ERL, the sweep rate was conducted. The influence of sweep rate on the electro-oxidation of ERL at OXL-9MGPE was investigated with varying a scan rate from 100-250 mVs^-1^ by recording the CVs (Figure 5a). The electrochemical mechanism can be assumed from the effect of sweep rate and to evaluate whether the process on the electrode is diffusion or adsorption controlled. Figure 5b reveals that with an increase in scan rate from 100-250 mVs^-1^, the peak current also increased. The graph of I_pa_ versus scan rate shows the straight line. The equation of linear regression is constructed as I_pa_ (µA) = 2.8892+ 0.0413 *v* (mV/s) (R^2^ = 0.9922), which suggest that the reaction at the electrode is adsorption controlled phenomenon. A graph of E_pa_ vs. log of scan rate provides a linear line (Figure 5c), the linear regression equation is constructed as E_pa_ (mV) = 397.48+ 89.53log*v* (mVs^-1^) with R^2^ 0.9875. According to laviron equation, for an irreversible process, E_p_ is defined by the equation, E_pa_ = E^0^ + (2.303 RT/αnF) log *v.* Where E^0^ is the redox potential, α is the electron transfer coefficient (α = 0.5 for the irreversible process), n is the number of electrons transferred and other symbols have their standard definitions.^27^ During the oxidation reaction, the transferred number of electrons can be calculated using the Laviron equation. E_pa_ vs. log *v* was plotted, from the graph slope is 89.53. The calculated number of an electron is 1.3 ≈ 1. This result provides that one electron transferred during the electro-oxidation of ERL. The graphical plot of log sweep rate vs. log I_pa_ (Figure 5d) was generated and the linear fitted equation is depicted as log I_pa_ (µA) = -0.636+0.7338 log *v* (R^2^ ꞊ 0.9993). The acquired slope from the constructed graph is 0.73 that is near to the hypothetical value 1.0 for adsorption-controlled processes.^28^ Therefore, the ERL electro-oxidation at OXL-9MGPE is an adsorption-controlled process.

### Evaluation of the pH effect on the electrochemical response of ERL at OXL-9MGPE

**Figure 6:**
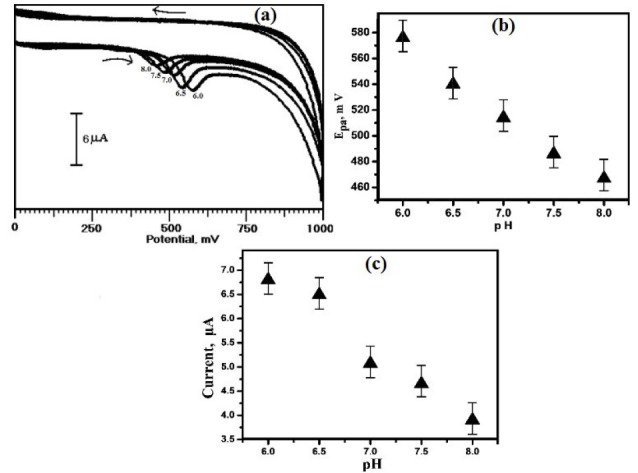
(a) CVs obtained using the OXL-9MGPE for the voltammetric response of 1×10^-4^ M ERL changing with pH from 6.0-8.0, at the potential scan rate 100 mVs^-1^, (b) The graphical plot of E_pa_ vs. pH, (c) The plot of I_pa_ vs. pH.

The pH of the supporting electrolyte significantly affects the voltammetric behaviour of ERL by shifting the potential towards the positive and negative. The CVs showing the influence of variation of pH on the electrochemical behaviour of ERL at OXL-9MGPE in 0.2 M PBS was deliberated with different pH from 6.0 to 8.0 by CV technique, which is expressed in Figure 6a. The peak potential was shifted to a less negative value, with the increase in pH of the solution (Figure 6b). The linear regression equation is represented as E_pa_ (mV) = 923.4 - 58.4 pH. The slope attained from the plot of pH vs. E_pa_ is 58.4 mV/pH, which is very near to the ideal value 59 mV/pH, specifies that the number of transferred electrons and protons are equal which is participated in the electrochemical reaction of ERL.^29^ The plot of pH vs. I_pa_ (Figure 6c), reveals that the highest peak current obtained at pH 6.0. Therefore, for the analysis of ERL, pH 6.0 was chosen for further experiments.

### Evaluation of effect of ERL concentration

**Figure 7:**
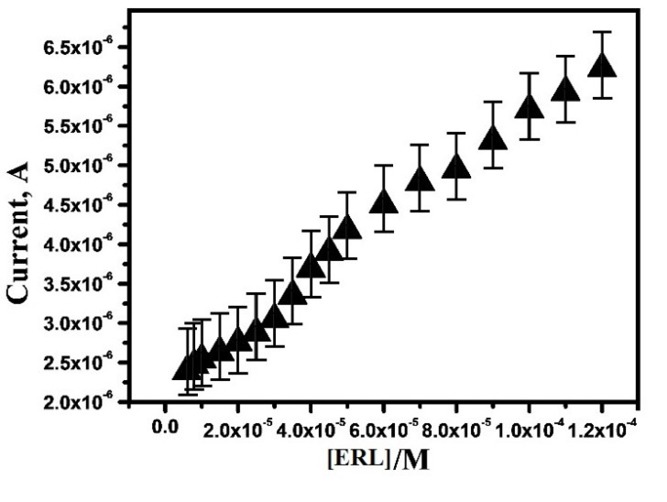
The graphical plot of I_pa_ vs. concentration of ERL.

The equipped sensor has the capability to detect ERL in lower quantity, with high sensing action is one of the main goals of this work. To examine the detection of low concentration of ERL, the electrochemical oxidation of ERL was carried out by various concentrations from the range 6 ×10^-6^ to 1.2×10^-4^ M at OXL -9MGPE in 0.2 M PBS of pH 6.0, at the sweep rate 100 mVs^-1^ by CV technique. Moreover, with intensification in ERL concentration, the anodic peak current increased. The plot of concentration of ERL vs. anodic peak current (Figure 7), which illustrates the linear relationship between the range of 4×10^-5^ to 1.2×10^-4^ M. The linear regression equation for calibration curve is I_pa_ (µA) = 2.55×10^-6^+ 0.0308 C (M), with the correlation coefficient 0.99. By using the formulae, the limit of detection (LOD) = 3SD/S; LOQ = 10SD/S, LOD and LOQ were calculated where SD is the standard deviation of 5 blank measurements, and S is the calibration plot slope.^30^ The LOD and LOQ estimated as 1.4×10^-6^ M and 4.7×10^-6^ M, respectively. The developed method was compared with the up to date reported method by the LOD value and the proposed electrode shows the LOD value which is close to the previous methods and tabulated in the Table 1.^31-34^

**Table 1:** Comparison of linear range and detection limits for ERL with previous methods and electrodes

**Method**	**Electrode**	**Linear working range (mol L** ^-1^ **)**	**Detection Limit (mol L** ^-1^ **)**	**Reference**
Amperometry	MWCNT/SPCE	1.0×10^-6^-1.0×10^-3^	5.3×10^-7^	31
SWV	BDD	2.0×10^-7^-2.0×10^-5^	1.7×10^-7^	32
CV	Ni-GCE	5.0×10^-6^-1.0×10^-4^	1.0×10^-6^	33
SWV	Pt/MWNTs/GCE	1.0×10^-6^ - 7.5×10^-5^	6.2×10^-7^	34
CV	OXL -9MGPE	4×10^-5^- 1.2×10^-4^	1.4×10^-6^	This work

MWCNT: multi-walled carbon nanotubes; SPCE: screen-printed carbon electrode; SWV: square- wave voltammetry; BDD: Boron-doped diamond electrode; GCE: glassy carbon electrode; CV: cyclic voltammetry; Ni: nickel; Pt/MWNTs/GCE: Pt-nanoclusters/ multi-walled carbon nanotubes.

### Evaluation of repeatability, reproducibility, and stability of OXL-9MGPE

To develop a good OXL-9MGPE, for the detection of ERL in 0.2 M PBS of pH 6.0, repeatability, reproducibility, and stability were scrutinized by utilizing the CV technique. Repetitive five measurements were conducted for the repeatability of OXL-9MGPE with a relative standard deviation 2.42%, which shows that OXL-9MGPE has the advantage of good repeatability. Five repetitive measurements were conducted for the reproducibility of OXL-9MGPE by changing the modified electrode and obtained relative standard deviation was 4.94%. This endorses that the modified electrode has the advantage of worthy reproducibility.

The stability of the OXL-9MGPE has been studied by 40 consecutive cycles. With the equation, % degradation = (I_pn_ /I_p1_) × 100, degradation percentage was assessed where I_pn_ and I_p1_ are the n^th^ and first cycle oxidation peak current respectively.^35^ It was observed that 95.87% of initial current response retained even after 40 cycles. This specifies that the OXL-9MGPE maintained its working condition even after 40 cycles. This indicates that there is good stability in the modified electrode.

### Investigation of electrochemical response of ERL at OXL-9MGPE and BGPE by DPV technique

**Figure 8:**
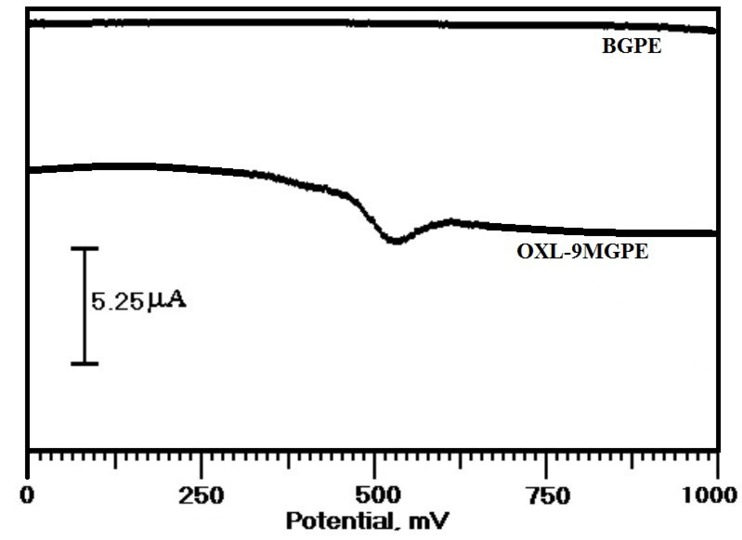
DPVs obtained for the voltammetric response of 1×10^-4^ M ERL at BGPE and OXL-9MGPE in 0.2 M PBS of pH 6.0, at the scan rate 50 mVs^-1^.

The voltammetric response of ERL at OXL-9MGPEwas examined by using the DPV technique. Figure 8 displays the differential pulse voltammograms (DPVs) of the electrochemical behaviour of ERL at OXL-9MGPEin 0.2 M PBS of pH 6.0, within the potential window from 0 to 1000 mV at the sweep rate of 50 mVs^-1^ by using DPV. The experimental condition for DPV were, pulse amplitude: 20 mV, pulse width: 60 m/s, pulse interval: 200 m/s. At OXL-9MGPE, a well-defined oxidation peak has appeared. The E_pa_ and I_pa_ were recorded at 528 mV and 10.24 µA, respectively. At BGPE, no peak was obtained. This indicates that the OXL-9MGPE enhanced the electro-oxidation of ERL by using the DPV technique.

### Simultaneous separation of ERL, DAN, URA by CV and DPV method at OXL-9MGPE

**Figure 9:**
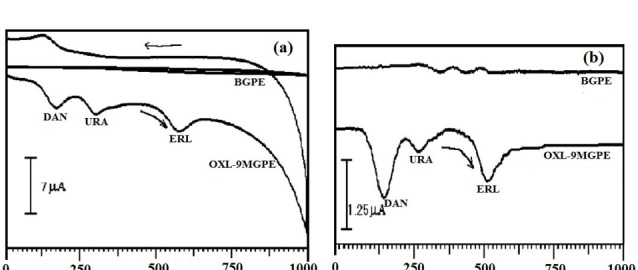
a) CVs of voltammetric response of ERL (1×10^-4^ M ), DAN (1×10^-4^ M ), and URA (1×10^-4^ M ) at BGPE and OXL-9MGPE in 0.2 M PBS of pH 6.0, at the scan rate 100 mVs^-1^, (b) DPVs of voltammetric response of ERL (1×10^-4^ M), DAN (1×10^-4^ M) and URA (1×10^-4^ M) at BGPE and OXL-9MGPE in 0.2 M PBS of pH 6.0, at the sweep rate 50 mVs^-1^.

The determination of ERL, DAN, and URA is performed simultaneously by using by CV approach.Figure 9a describes the CVs of electrochemical response of ERL along with the DAN, and URA at OXL-9MGPEin 0.2 M PBS of pH 6.0. The concurrent detection of ERL (1×10^-4^ M), DAN (1×10^-4^ M) and URA (1×10^-4^ M) were performed and E_pa_ noticed at 576 mV, 172 mV, and 305 mV respectively. At BGPE no peak had appeared. The above result indicates that OXL-9MGPEshows good selectivity towards the electrochemical response of ERL with DAN and URA by the CV method.

Instantaneous detection of ERL, DAN, and URA was further performed by using the DPV technique. The Differential voltammograms were recorded for the electro-oxidation of ERL along with DAN and URA at BGPE and OXL-9MGPEin 0.2 M PBS of pH 6.0, with the potential window from 0 to 1000 mV at the sweep rate of 50 mVs^-1^(Figure 9b). A well-separated peak was observed for ERL, DAN, and URA. The peak potential of ERL, DAN, and URA was found at 510 mV, 141 mV and, 267 mV, respectively. At BGPE, the low voltammetric response was obtained. Thus, OXL-9MGPEshows an excellent selectivity towards the electro-oxidation of ERL in the interference of DAN and URA by DPV technique.

## Conclusion

In this current voltammetric studies, construction of an electrochemical sensor with OXL-9MGPE for the analysis of ERL in 0.2 M PBS of pH6.0, CV and DPV strategy was successfully utilized. With the boosted current response, very low level of detection and high sensitivity, the OXL-9MGPE has an outstanding efficiency in electro-oxidation of ERL. The proposed sensor showed good stability, excellent reproducibility, and, adequate repeatability. Furthermore, the interference study of ERL with DAN and URA was carried out, and the results indicate an excellent selectivity of the proposed sensor. Finally, for the ERL assessment, a voltammetric technique has been established. The proposed method has been applied effectively to determine ERL.

## Ethical Issues

No ethical issues for this present work.

## Conflict of Interest

The authors declare no conflict of interest for this work from any organization, authors and reviewers.

## Acknowledgments

We gratefully acknowledge the financial support from the VGST, Bangalore under Research Project. No. KSTePS/VGST-KFIST (L1)2016-2017/GRD-559/2017- 18/126/333, 21/11/2017 and SC/ST Fellowship No. MU/SCT RF/CR17/2017-18, Mangalore University.
